# The roles of intratumour heterogeneity in the biology and treatment of pancreatic ductal adenocarcinoma

**DOI:** 10.1038/s41388-022-02448-x

**Published:** 2022-09-10

**Authors:** Theodore Evan, Victoria Min-Yi Wang, Axel Behrens

**Affiliations:** 1 Cancer Stem Cell Laboratory, The Breast Cancer Now Toby Robins Research Centre, Institute of Cancer Research, London, SW3 6JB UK; 2 CRUK Beatson Institute, G61 1BD Glasgow, UK; 3 Department of Surgery and Cancer, Imperial College London, London, SW7 2AZ UK; 4 CRUK Convergence Science Centre, Imperial College London, SW7 2AZ London, UK

**Keywords:** Tumour heterogeneity, Cancer stem cells, Cancer metabolism, Pancreatic cancer

## Abstract

Intratumour heterogeneity (ITH) has become an important focus of cancer research in recent years. ITH describes the cellular variation that enables tumour evolution, including tumour progression, metastasis and resistance to treatment. The selection and expansion of genetically distinct treatment-resistant cancer cell clones provides one explanation for treatment failure. However, tumour cell variation need not be genetically encoded. In pancreatic ductal adenocarcinoma (PDAC) in particular, the complex tumour microenvironment as well as crosstalk between tumour and stromal cells result in exceptionally variable tumour cell phenotypes that are also highly adaptable. In this review we discuss four different types of phenotypic heterogeneity within PDAC, from morphological to metabolic heterogeneity. We suggest that these different types of ITH are not independent, but, rather, can inform one another. Lastly, we highlight recent findings that suggest how therapeutic efforts may halt PDAC progression by constraining cellular heterogeneity.

## Introduction

Pancreatic ductal adenocarcinoma (PDAC) is the most common and most aggressive type of pancreatic cancer with a five-year survival rate of only 8% [1]. Although PDAC incidence is low compared to that of breast, prostate and lung cancers, it is projected to become the second-most common cause of cancer-related deaths, after lung cancer, by 2030 [2]. This trend is partly attributable to a doubling of pancreatic cancer incidence between 1990 and 2017 due to growing populations, increased life expectancy and rising incidence of risk factors [3–5].

The high proportion of metastatic disease at diagnosis is one of the main reasons for the high mortality rate of PDAC. The best curative treatment option is surgery, but even the small proportion of patients eligible for surgery has a five-year survival rate of only 30% [1]. Both adjuvant and neo-adjuvant chemotherapies typically cannot halt disease progression for more than a few months. There are currently no targeted therapies for PDAC due to its near-universal reliance on “undruggable” versions of mutant KRAS [6]. Only 1–3% of PDAC tumours contain the now-druggable KRAS^G12C^ mutation [7], an inhibitor of which is showing great promise in KRAS^G12C^-mutated non-small cell lung cancer [8].

One reason for the poor chemotherapeutic response of PDAC tumours is their cellular heterogeneity. Whereas inter-tumour heterogeneity (differences between PDAC in different patients) can in principle be managed by careful classification of tumour subtypes and identification of subtype-specific vulnerabilities, intratumour heterogeneity (ITH) increases the likelihood that a subset of PDAC cells will be resistant to a given treatment, thwarting attempts at precision medicine. Genetic ITH, the existence of genetic subclones within the tumour of a single patient, was first demonstrated in PDAC by karyotyping [9] and later by DNA sequencing [10, 11]. However, there is compelling evidence to suggest that these genetic subclones can only explain a proportion of the phenotypic heterogeneity observed among cancer cells.

In this review, we discuss four types of ITH observed in PDAC—cancer “stemness”, transcriptional and epigenetic variation, the epithelial-to-mesenchymal spectrum, and metabolic differences. These phenotypic variables have all been associated with the aggressive and chemoresistant nature of PDAC. As we describe, these types of tumour cell heterogeneity are partly overlapping and often linked, although in many cases the triggers influencing the adoption of different cell states are not known. There is still much to discover about the impact of each variable on tumour progression, but recent studies have begun to dissect the drivers of heterogeneity itself, with the aim of reducing the complexity of PDAC to render it more clinically tractable.

## Cancer stem cells (CSCs): reservoirs of tumour-initiating capacity

Like normal stem cells present in almost all organs, CSCs are defined by the two properties of self-renewal—the ability to give rise to more CSCs—and regenerative capacity—the ability to give rise to non-stem cells to recapitulate the histology of the tissue or tumour. In the case of PDAC, this usually results in a combination of epithelial and mesenchymal tumour cells, together with recruitment of stromal cells. CSCs therefore give rise to two types of ITH: cells differing in their tumour-initiating capacity (cancer stem versus non-stem cells) and cells arising from CSCs that differ in morphology and behaviour.

The CSC model was first applied to leukaemia stem cells, with the term “cancer stem cell” coined in 2001 [12], and CSC markers were rapidly identified in several solid cancers, including PDAC [13–16]. Perhaps because there is no clearly defined stem/progenitor hierarchy for the healthy pancreas, unlike the intestine for example, there has as yet been little consensus on PDAC CSC markers. The first tumour-initiating cells identified in primary human PDAC samples were marked by the cell surface markers CD133 and CXCR4 in one study [15], and the combination of CD44, CD24 and EpCAM in a second study [14]. A host of other markers and metabolic activity assays—c-MET [17], aldehyde dehydrogenase activity [18, 19], tuft cell-like cells marked by Dclk1 and acetylated tubulin [20, 21], ABCG2-dependent riboflavin accumulation and its endogenous fluorescence [22], CD90 [23], Msi1 and Msi2 [24], and CD9 [25]—have also been reported to enrich for CSCs. Taken together these experiments suggest that there are PDAC cancer cell subpopulations that are enriched for tumour-initiating capacity. Clonal analysis using genetic barcoding in primary human PDAC samples supports this view [26, 27]. However, not all cells positive for a given marker will be bona fide CSCs.

Traditional conceptions of the CSC theory assume that cell-intrinsic mechanisms maintain a strict hierarchy of CSCs and non-CSCs [16]. An important corollary of this theory is that if CSCs are not ablated, cancers will always relapse, and conversely, that therapeutic targeting of CSCs should lead to durable therapeutic responses. However, it has become clear from research in the intestine that “one-shot” targeted ablation of CSCs in solid tumours may not be definitive, because non-CSCs can assume CSC properties, upending the traditional hierarchy [28, 29]. The latest research has therefore focused on the molecular determinants of CSC properties such as tumour-initiating capacity, and on a “functional hierarchy” of stemness within the tumour, rather than on markers of a hard-wired CSC identity [30].

The mRNA-binding proteins Msi1 and Msi2 have been most comprehensively characterised in murine PDAC as CSC markers that also enhance tumour-initiating capacity. As an mRNA-binding protein, Msi2 directly binds and modulates the levels of mRNA molecules coding for epigenetic modifiers such as Brd4 and Hmga2 [31], explaining, at least in part, how Msi2-expressing cells are highly tumourigenic. In a follow-up study, the same group characterised Msi2-expressing CSCs through a multi-omic approach [24], identifying upregulation of lipid and redox metabolic pathways as distinguishing features of CSCs. Furthermore, Msi2-expressing CSCs have a different epigenetic landscape compared to the bulk of PDAC cells. One of the highly expressed transcription factors in Msi2-expressing CSCs is the nuclear hormone receptor RORγ, which controls both stemness and proto-oncogenic transcriptional programs and could be pharmacologically targeted to reduce tumour burden in human and murine models. Msi1/2 knockout also increases survival in the widely-used KPC (LSL-KRas^G12D^; Trp53^fl/fl^ or Trp53^fl/+^; Pdx1-Cre) mouse model of PDAC. Although these mice eventually succumb to the disease, it is possible that Msi deletion is incomplete or compensated for by the other homologue. Even considering this caveat, Msi-deleted tumours show a more epithelial histology than their KPC counterparts [31].

**Fig. 1:**
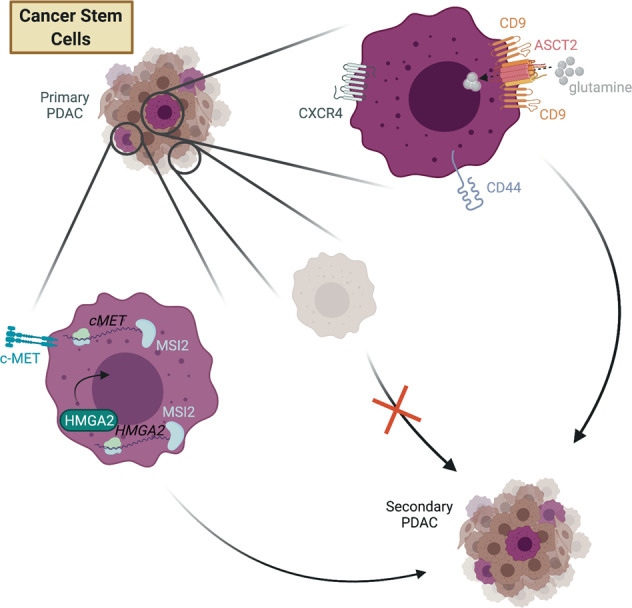
Only a subset of PDAC cells—the CSCs—have tumour-initiating capacity. CSCs are marked by a variety of cell surface markers and/or transcriptional programs.

More recently our laboratory identified the cell surface tetraspanin CD9 as a marker for both murine and human PDAC CSCs [25]. CD9^high^, but not CD9^low^, PDAC cells re-initiate tumour grafts that resemble the mixed—epithelial and mesenchymal—histology of primary KPC tumours. Mechanistically, CD9 interacts with the glutamine transporter ASCT2 and thus boosts glutamine import and downstream metabolism in CSCs. Heterozygous CD9 deletion in the KPC mouse model extends lifespan, suggesting that the facilitation of glutamine metabolism is an important function of CD9 in an autochthonous PDAC model. Several other studies also suggest that glutamine metabolism is critical to PDAC progression [32–35], underlining enhanced glutamine metabolism as a feature of CSCs and a driver of tumour heterogeneity. Figure 1 depicts how CSCs and non-CSCs differ in their tumour-initiating capabilities.

Cross-species validation of CSC markers has sometimes yielded conflicting results. In mouse PDAC, some of the previously described human PDAC CSC markers including CD133, CD44, CD24 and ALDH1 activity fail to distinguish tumourigenic from non-tumourigenic cell populations [36]. Studies carried out in mouse models of PDAC indicate that the frequency of cells with tumour-initiating capacity is, in general, much higher than in human samples, possibly for technical reasons such as increased viability upon cell sorting, or the use of same-species engraftment assays to test tumour-initiating capacity [36]. Use of a variety of mouse, patient-derived xenograft and organoid models should help to disentangle these possibilities.

Taken together, the results from the studies described above point towards a CSC theory in PDAC in which there is no one fixed CSC subpopulation that is intrinsically hard-wired to act as a tumour-initiating population. Instead, there is a heterogeneously distributed CSC “state” within a given tumour [30, 37–39]. Despite this more functional definition, there remains great interest in CSCs as drivers of tumour progression, metastasis and relapse.

An ideal anti-CSC therapy would prevent tumour cells from acquiring CSC capacity by steering all cells away from the stem-like state, but in practice, targeting existing CSCs via specific markers may be sufficient to reduce tumour heterogeneity and render PDAC more treatable. Given the data from a colorectal cancer model showing that metastases are more reliant on Lgr5^+^ CSCs than primary tumours [29] it will be interesting to see if PDAC primary tumours and metastases react differently to CSC ablation, especially given that metastases are the main cause of patient mortality [40]. In the case of Msi2/RORγ-expressing CSCs there is some promise that the CSC transcriptional state could be therapeutically targeted; it remains to be seen if this will be the case for other highly plastic aspects of stemness in PDAC.

## Transcriptional and epigenetic heterogeneity: determining cell state

Transcriptional subtypes of PDAC have proven considerably more robust than genetic driver mutations in classifying patients into relevant disease groups. Regulation of gene expression is multifaceted and may occur at both genetic and epigenetic levels. The epigenome—the myriad modifications that drive differences in gene expression independently of changes in DNA sequence—includes processes to silence or activate genes at the level of chromatin accessibility, histone modifications, DNA methylation and non-coding regulatory RNAs [41, 42]. The role of the epigenome itself as a potential driver of PDAC has been highlighted by recent large-scale DNA sequencing studies, which have identified frequent mutations in epigenetic modifiers, including SWI/SNF components such as ARID1A and SMARCA4, the demethylase KDM6A, and acetyltransferase EP300 [43, 44]. The pathogenicity of some of these genes has been validated in genetically engineered mouse models [45, 46]. Differences in both DNA methylation and chromatin modification patterns correlate with distinct PDAC subtypes in patient-derived xenografts (PDXs), suggesting that distinct epigenetic states may underpin inter-patient PDAC transcriptional heterogeneity [47]. The “squamous” morphological and transcriptional subtype of PDAC has consistently been associated with the poorest outcomes in multiple studies [43, 46, 48].

**Fig. 2:**
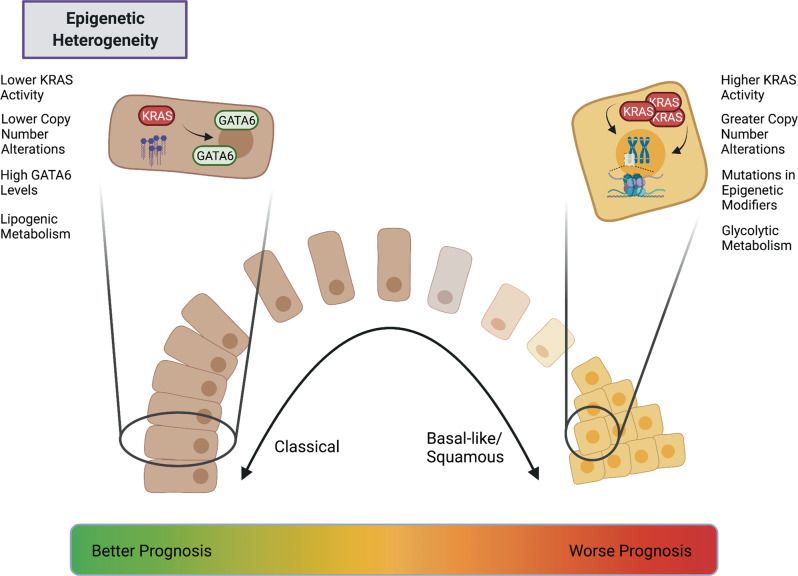
Both classical and squamous-like PDAC cells—defined by different transcriptional and epigenetic programs—can be found within a single tumour. Patients whose PDACs exhibit predominantly classical features have a better prognosis than those whose tumours exhibit predominantly squamous features, such as higher KRAS activity and mutations in epigenetic modifiers.

However, in many patient tumours, “squamous” morphology is observed heterogeneously within tumours, suggesting that these subtypes are not fixed but rather an emergent property of PDAC evolution. Intriguingly, tumours with subclonal mutations in chromatin modifiers such as KDM6A and ARID1A are more likely to exhibit histological features of the squamous subtype, suggesting this broad family of mutations predispose PDAC cells to adopt poor prognostic features [49]. Figure 2 highlights some of the differences between the “classical” and squamous-like PDAC cells.

One area in which epigenetic heterogeneity has been unequivocally demonstrated within individual patients is when comparing primary tumours and their metastases. Studies of paired PDAC samples from primary tumours and metastases, in both mouse and human rapid-autopsy specimens, have identified broad epigenetic reprogramming and changes in chromatin modifications strongly associated with metastasis. Analyses of the chromatin landscape of paired metastasis-derived and primary tumour-derived KP*C (LSL-KRas^G12D^; Trp53^R172H/+^; Pdx1-Cre) organoids implicates broad enhancer reprogramming as a mechanism imbuing PDAC cells with metastatic competence [50]. This enhancer reprogramming leads to increased binding of a select group of transcription factors, notably FoxA1. Analogous work with paired rapid-autopsy specimens from human patients has also identified considerable changes in the chromatin landscape in metastatic cells. This study demonstrated that a subset of metastatic lesions evolves a unique metabolic dependency on the oxidative pentose phosphate pathway, uncovering a link between metabolic and epigenetic changes in metastasis, and suggesting that such changes may, in some circumstances, be therapeutically tractable [51]. In these studies, it is not clear whether the changes observed in metastatic tumour cells are already present heterogeneously in the primary tumour, or whether they are specific to the secondary metastatic setting. In contrast, a transient, highly metastatic subpopulation can be identified in KP*C tumours using a fluorescent reporter for the transcriptional regulator Hmga2 [52]. These cells are defined by a hypoxic gene signature driven by the transcription factor Blimp1, a signature also associated with poor outcomes in human disease. Collectively, these experiments argue that both transcriptional and epigenetic changes may facilitate metastasis, but critically, they do not uncover a single unified mechanism, suggesting that metastatic competence can arise in multiple ways.

The premise of specifically targeting transcriptional or epigenetic drivers of disease progression is a considerable challenge. Current compounds targeting the epigenome lack specificity [42, 53], which may explain their limited clinical efficacy in solid tumours. Therefore, it is reasonable to ask what stimuli drive transcriptional changes during tumour evolution, and whether these can be targeted. In the case of Blimp1 expression [52], the hypoxic microenvironment was identified as such a stimulus. However, genetic strategies that render the PDAC microenvironment less hypoxic can generate more aggressive, poorly differentiated tumours in other PDAC models [54], emphasising the complexity of targeting specific cell states without considering potential secondary effects. A detailed evaluation of how clinically relevant transcriptional states arise will allow more informed approaches to targeting the transcriptome and epigenome.

One such plausible mechanism is through genomic instability. Initial genetic reconstruction efforts in human PDAC failed to identify somatic mutations unambiguously associated with metastasis [11]. More recent, comprehensive genomic sequencing demonstrated limited genetic diversity between human metastatic samples and the paired primary PDAC, further suggesting that metastatic competence is not driven by novel, metastasis-specific driver mutations [55]. However, these studies have historically focused on single-nucleotide variants and as a result discounted the potential impact of copy number variations as genetic drivers. In addition to being the most frequently mutated oncogene in PDAC, gene dosage increases of mutant *KRAS* are already prevalent at early stages of tumour formation, and also drive metastatic dissemination in both mouse and human [56]. More recent evidence confirms that both localised copy number variations in *KRAS* and genes encoding epithelial-mesenchymal transition-related transcription factors such as *GATA6*, as well as whole-genome doubling events, may independently drive squamous-like transcriptional and morphological changes in human PDAC cells [57]. The extent to which these changes occur heterogeneously within precursor lesions and tumours, and how these populations might interact, remains to be investigated. Data from intraductally transplanted orthotopic xenografts using patient-derived organoids also implicates *KRAS* amplification in promoting squamous-like, invasive features within heterogeneous tumours, since inducible genetic activation of KRAS promotes an invasive phenotype in these models [58]. Given that mutations in epigenetic modifiers are also associated with squamous differentiation [49], it is tempting to hypothesise that these mutations promote genomic instability or bias it towards an evolutionary trajectory favouring amplification of the KRAS signalling pathway. Whether this can one day inform subtype-specific therapeutic strategies remains to be seen, though recent work suggests that whole-genome doubled cancer cells exhibit specific vulnerabilities that may be amenable to targeting [59].

## The epithelial-mesenchymal spectrum: the tumour as a chronic wound

The epithelial-mesenchymal transition (EMT) is a dynamic, reversible process in which epithelial cells lose their local attachments and apical-basal polarity to adopt a spindle-shaped morphology and increased motility [60]. This morphological and functional change in cellular phenotype manifests as a loss of expression of canonical epithelial proteins, such as E-cadherin, coupled to a gain of mesenchymal proteins, such as vimentin, N-cadherin and FSP1 [61]. EMT programs are activated by a wide variety of stimuli, notably cytokines such as TGF-ß [62], hypoxia [63], matrix stiffness [64] and varied stromal cell inputs [65].

Intense research interest in EMT has been driven in part due to its association with invasion and metastasis. In the KPC model, mesenchymal-like cells can be identified in the earliest stages of pancreatic cancer, even preceding the formation of a defined primary tumour [66]. These mesenchymal cells can colonise the liver and other distant organs, lending credence to the high metastatic capacity of cells that have undergone EMT. In mice, KRas^G12D^-mutant pancreatic epithelial cells spontaneously escape replicative senescence to generate two distinct tumour cell phenotypes—one epithelial and the other mesenchymal—each with distinct molecular drivers for survival and proliferation [67]. Mesenchymal tumour cells in this model are more metastatic and aggressive but highly sensitive to inhibitors of proteostasis, linking EMT to disease severity while emphasising the potential therapeutic avenues opened through characterising this type of ITH.

The significance of EMT in PDAC and other solid tumours in humans has been historically more controversial [68]. Nonetheless, histological studies employing pathological scoring criteria to distinguish cancer and stromal cells have observed a correlation between increased expression of EMT-related transcription factors in tumour cells and poorer clinical outcomes [69, 70]. Detection of circulating tumour cells with mesenchymal features is also associated with poor prognosis in PDAC [71]. Furthermore, functional studies have demonstrated that induction of EMT genes correlates with the aggressive squamous/basal-like PDAC transcriptional signature [72] in both murine models [73] and patient-derived organoids [74], suggesting a relationship between this type of ITH and transcriptional subtypes correlated with distinct patient outcomes.

EMT programmes are orchestrated by a group of transcription factors, the EMT-TFs, of which TWIST1, ZEB1/2 and SNAI1/2 are the most comprehensively studied [75], though other transcription factors may also promote EMT in specific contexts [76]. However, targeted genetic deletion strategies have shown that these EMT-TFs are functionally distinct in PDAC, with only a subset significantly promoting ITH. Solitary genetic ablation of Snail or Twist1 does not significantly alter the cellular composition of PDAC tumours in murine models, though does render tumours more chemosensitive [77]. Conversely, ablation of Zeb1 in KP*C tumours results in a dramatic histological and functional phenotype wherein tumours are rendered almost entirely epithelial, with significantly reduced metastatic burden [78]. Zeb1 is also reported to inhibit the expression of stemness-repressing miRNAs in PDAC cells, suggesting this loss of heterogeneity may also be linked to a loss of CSC properties in the tumour [79]. However, despite limited metastatic burden, KP*C Zeb1 knockout mice have no significant survival benefit, likely because growth of the the primary tumour was not constrained. This is consistent with EMT promoting invasion and metastasis but not necessarily restraining proliferation.

We recently demonstrated that an interaction between the epithelial and mesenchymal subpopulations regulates EMT in PDAC. We found that the BMP inhibitor GREM1 is highly expressed in mesenchymal subpopulations of KPC tumours and acts in a paracrine manner to suppress EMT in the epithelial subpopulation, by reducing expression of the EMT-TFs *Snail* and *Slug*. Deletion of *Grem1* leads to an almost complete switch of cancer cells to a mesenchymal phenotype, with an associated increase in metastatic frequency. Thus, a single soluble factor, GREM1, ensures the co-existence of both epithelial and mesenchymal PDAC subpopulations [80].

**Fig. 3:**
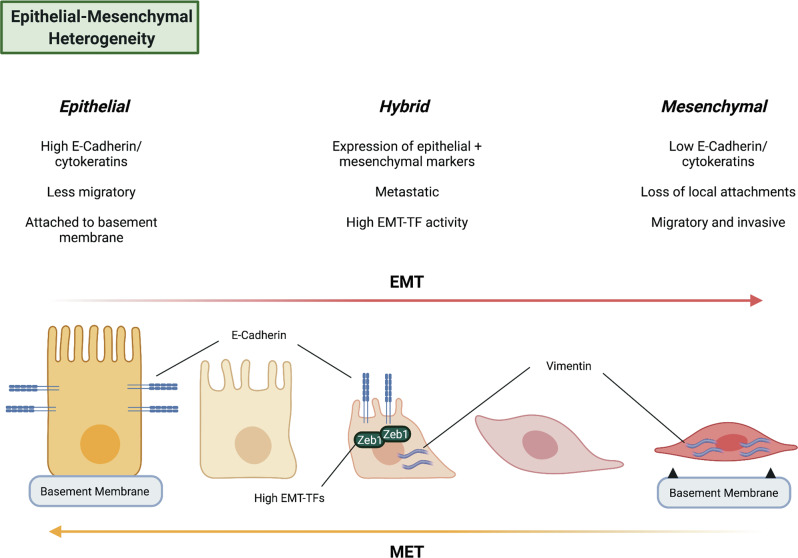
PDAC cells along the EMT spectrum express different markers and exhibit different behaviours. In particular, recent work highlights the existence of hybrid or partial EMT states that combine features of epithelial and mesenchymal tumour cells.

More nuanced prospective isolation strategies and the application of single-cell technologies have further complicated the classical binary model of EMT. Recent evidence, from multiple tissue and tumour types, supports “partial EMT” states, which exhibit transcriptional characteristics of both epithelial and mesenchymal cells [81]. These partial EMT states also exist in PDAC. Aiello et al. [82] utilised a sorting strategy in KPC tumours based on membrane expression of E-cadherin to identify a partial EMT population in a subset of murine PDACs, in which mesenchymal genes are upregulated in the absence of concurrent downregulation of canonical epithelial genes. Importantly, the partial EMT state in this study was associated with collective migration behaviour, whereas the complete EMT state was associated with single-cell invasion, demonstrating that partial and complete EMT are functionally distinct. Recent single-cell RNA sequencing studies in murine PDAC have supported these conclusions, suggesting that EMT phenotypes become more common as the disease progresses and that they manifest across a transcriptional spectrum [83]. A separate single-cell lineage tracing study using a CRISPR-Cas9-based approach has further highlighted that such hybrid cells are highly metastatic relative to fully committed mesenchymal or epithelial clones, at least in the KP*C model [84]. This idea is consistent with a recent comprehensive mapping study of EMT transition states in murine skin and breast tumours [85], which similarly found different invasive, metastatic and tumourigenic capacities for these populations across the EMT spectrum. This variety of EMT phenotypes has recently been characterised in human PDAC samples as well [86, 87], suggesting that such transition states are not simply an artefact of murine models and may have therapeutic relevance. Figure 3 shows various features of PDAC cells along the EMT spectrum.

Nonetheless, there are several unresolved questions related to EMT and PDAC. The majority of PDAC metastases in both genetically engineered mouse models and human patients have an epithelial histology [88, 89], and there is evidence that stabilisation of an epithelial phenotype may actually promote metastasis in certain contexts [87, 89]. However, mesenchymal cells can also undergo EMT reversal. This process, termed mesenchymal-epithelial transition (MET), has been described in other tumour types and murine PDAC [88, 90, 91]. Furthermore, loss of the canonical EMT-TF Zeb1 in murine PDAC significantly reduces but does not eliminate PDAC metastasis [78], suggesting either compensatory effects from other EMT-TFs or the existence of EMT-independent mechanisms of metastasis. Epithelial and mesenchymal tumour cells in murine PDAC also have distinct metastatic organotropism [89], further complicating the idea that all metastases require an EMT. Finally, EMT transcriptional signatures are not a feature of all metastatic cells characterised in murine models and human patients [50–52]. Currently, the collective sum of evidence favours EMT as one of a number of mechanisms by which pancreatic cancer cells achieve invasive and metastatic competence. Future research will refine this picture.

## Metabolic heterogeneity: differing demands for survival

The fact that tumour metabolism differs from that of its organ of origin was first recognised almost a century ago by Otto Warburg. Solid tumours are generally more glycolytic than healthy tissues even when oxygen is not limiting [92]. This aerobic glycolysis, now known as the Warburg effect [93], is one of the dysregulated metabolic pathways that contributes to “reprogramming cellular energetics”, an emerging hallmark of cancer [94]. Increased dependence on and metabolism of the conditionally essential amino acid glutamine is another such pathway [32, 33].

Constitutively active mutant KRAS, which drives over 90% of all PDAC cases [48], is a major genetic driver of metabolic dysregulation in PDAC [95–97]. However, there is evidence for considerable inter-patient metabolic heterogeneity despite the overarching dominance of KRAS activation. For example, a large-scale study of human PDAC cell lines, almost all harbouring KRAS mutations, revealed three distinct metabolic subtypes: slow-proliferating, lipogenic and glycolytic [98]. Further analysis showed that these differences are driven, in part, by underlying differences in gene expression and protein abundances. The glycolytic and lipogenic subtypes are linked to the previously reported “quasi-mesenchymal”/“squamous” and “classical” transcriptional subtypes of PDAC, respectively [99, 100]. Metabolite profiling of PDXs in conjunction with transcriptional subtype classifications [43, 72] has confirmed a link between the “classical” subtype and lipid metabolism, which is independent of KRAS mutation and amplification status [101]. More recently, transcriptomic analyses of large PDAC patient cohorts also found an association between glycolytic tumours and the “squamous” subtype, and between cholesterogenic tumours and the “classical” subtype [102].

Intratumour metabolic heterogeneity is much more challenging to study for technical reasons, but given that differences in metabolic pathways have been described as potential drivers of the CSC phenotype in PDAC [24, 25], it is expected that differences in metabolism between tumour cells will be at least as great as variance in tumour-initating capacity. Imaging techniques that minimise the need for sample handling can yield an overview of metabolic activity in individual cells within organoids [103]. Drug-induced metabolic changes within primary human PDAC samples are not homogeneous [104]. Strikingly, patients whose organoids have a homogeneous metabolic response to treatment have recurrence-free survival exceeding a year, whereas those with no or heterogeneous metabolic responses exhibit recurrence within a year of surgery [105]. Though the number of patients tested in this study was small, this finding corroborates the idea that metabolic heterogeneity contributes to disease outcome. Alternatively, intratumour metabolic heterogeneity can be roughly inferred through transcriptional heterogeneity: the putative tumour suppressor *ISL2* is epigenetically silenced in only some regions of human PDAC tumours, and *ISL2* downregulation leads to enhanced expression of genes involved in oxidative phosphorylation, thus potentially rendering those cells sensitive to mitochondrial electron transport chain inhibitors [106].

Several studies have reported differences in metabolism between different CSC-enriched populations and the bulk of PDAC cells. Human PDAC cells grown as tumour spheres are less glycolytic than cells grown in adherent culture, relying more on oxidative phosphorylation, and are more sensitive to the mitochondrial electron transport chain inhibitor metformin. Tumour spheres are also better able to tolerate low glucose or low glutamine conditions [107]. However, it is unclear to what extent different cell culture conditions themselves in these experiments influence metabolic pathway usage. Another study found increased levels of autophagy in PDAC cells marked by ALDH activity and CD44/CD133 expression, with inhibition of autophagic flux decreasing the proportion of viable CSCs [108]. Interestingly, when mutant KRas expression is switched off in murine PDAC tumours, surviving cells rely on mitochondrial biogenesis, oxidative phosphorylation and autophagy for survival and re-initiation of tumour growth [109, 110]. Our recent work indicates that increased glutamine import and downstream use in the tricarboxylic acid cycle correlate with tumour-initiating properties of murine PDAC cells. PDAC CSCs marked by high levels of CD9 also have high levels of the glutamine transporter ASCT2 at the cell surface. CD9 knockout in murine PDAC cells decreases organoid formation capacity of these cells, but this can be rescued by supplementing excess glutamine or overexpressing ASCT2 [25]. Independently, another group has found that expression of a mitochondria-specific ASCT2 isoform is important for PDAC cell survival [111]. Together these experiments suggest that PDAC tumour-initiating cells rely more heavily on mitochondrial metabolism and autophagy than their non-stem cell counterparts, rendering CSCs potentially more vulnerable to inhibitors of these metabolic pathways.

Several findings highlight the metabolic plasticity of PDAC cells upon perturbation. As an example, whereas heterozygous CD9 knockout prolonged survival in KPC mice, homozygous CD9 knockout did not. Metabolomic profiling showed that homozygous CD9 knockout cells were more similar to CD9 wild-type cells than to heterozygous knockouts, indicating that homozygous knockout leads to compensatory metabolic rewiring to maintain high levels of glutamine metabolism [25]. Similar metabolic compensation has been observed in KPC mice treated with the glutaminase inhibitor CB-839 [112], further emphasising the metabolic plasticity of PDAC tumours [113, 114].

A common product of PDAC metabolism is lactate, which needs to be exported from cells, for example via monocarboxylate transporters (MCTs), to maintain intracellular pH in a physiological range. High levels of MCT4 mark highly aggressive, glycolytic tumours and human PDAC cell lines. However, MCT4 knockdown leads to upregulation of an alternative transporter, MCT1, as well as to compensatory mitochondrial oxidative phosphorylation and autophagy [115]. In the context of spatially heterogeneous tumours, lower availability of glutamine in the core, versus the periphery, of subcutaneous PDAC xenografts leads to enhanced uptake of extracellular nutrients via macropinocytosis in an effort to compensate this paucity [116]. Limiting levels of alanine in the PDAC microenvironment also create metabolic niches in which stromal and tumour cells exchange alanine via specific transporters [117]. The ability of cells to adjust and maintain their metabolism in the face of significant perturbations means that monotherapy targeting a single metabolic pathway is unlikely to be successful. Nonetheless, inhibition of tumour metabolism remains a promising goal, as combinatorial approaches that constrain metabolic heterogeneity may generate unforeseen therapeutic vulnerabilities [118]. This phenomenon has been demonstrated for lactate dehydrogenase inhibition in PDAC cell lines, which is most successful when combined with inhibitors of mitochondrial metabolism such that metabolic plasticity is compromised [119].

The gene encoding the tumour suppressor *SMAD4* is deleted in approximately 30% of PDAC samples [44]. A recent study has now implicated SMAD4 as a regulator of the glycolytic enzyme phosphoglycerate kinase 1 (PGK1), with *SMAD4* loss correlating with higher levels of PGK1 in patient samples and upregulation of PGK1 in human PDAC cell lines. Of note, PGK1 expression is heterogeneous across human PDAC samples, with both the total levels of the protein as well as its intracellular localisation varying, suggesting that within SMAD4-negative tumours there are differences in metabolic states. Higher levels of cytoplasmic PGK1 stimulate glycolysis, but PGK1 can also translocate into the nucleus to act as a transcriptional repressor of *CDH1* (the gene encoding the epithelial marker E-cadherin) [120]. How this link between the glycolytic and mesenchymal phenotypes fits with the canonical function of SMAD4 as a mediator of TGF-β signalling and the more epithelial character of SMAD4-deleted tumours [121] remains to be elucidated. In human PDAC cell lines, expression of the lactate exporter MCT4 also correlates with higher levels of vimentin expression and a more mesenchymal phenotype [115].

**Fig. 4:**
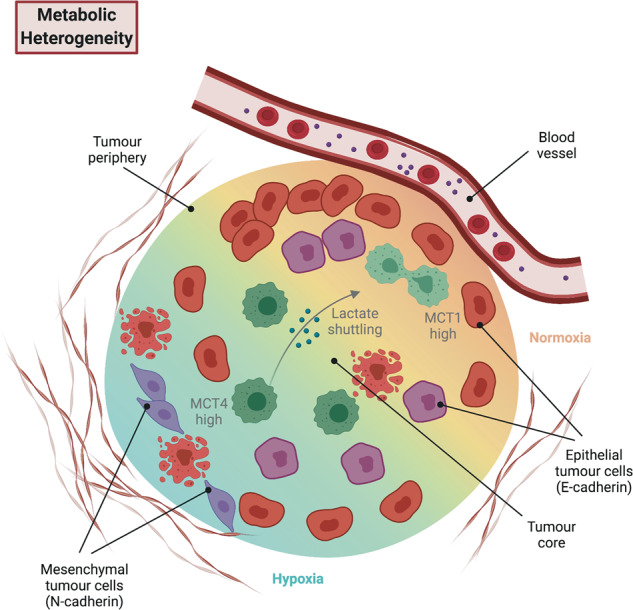
PDAC tumours contain spatially distinct metabolic zones. Metabolic heterogeneity and associated differences in metabolic pathways within tumours (e.g. lactate production vs. uptake) affect tumour cell phenotypes and therapeutic responses.

Solid tumours contain both normoxic as well as hypoxic regions depending on their proximity to intact blood vessels. These hypoxic regions are particularly pronounced in PDAC due to the extensive desmoplastic stromal reaction that surrounds pockets of tumour cells [122]. Furthermore, both reduced blood flow and extensive hypoxia correlate with poorer clinical outcome and metastasis formation [123, 124]. Whereas normoxic cancer cells use glycolysis to generate pyruvate, hypoxic cells produce lactate through anaerobic glycolysis. Thus, the tumour can be divided into metabolic “zones” based on their level of hypoxia, as has been described for glioblastoma [125]. More than ten years ago it was shown that lactate produced by hypoxic cells can be shuttled to more normoxic cancer cells to fuel oxidative phosphorylation in a symbiotic relationship. Hypoxic cells preferentially express MCT4 for lactate export whereas normoxic cells express MCT1 for lactate import [126]. This symbiotic lactate shuttle has been observed in a mouse model of PDAC [63]. Lactate can also be transferred from more hypoxic to normoxic PDAC cells via connexin shuttles [127], demonstrating that tumour cells can cooperate to manage local metabolic constraints. Figure 4 highlights these metabolic zones and how lactate might be moved between them.

High rates of lactate production and extrusion into the extracellular environment can lead to chronic acidosis, which in turn leads to oxidative stress [128]. To combat this oxidative stress, PDAC cells shuttle glutamine into a non-canonical cytoplasmic metabolic pathway driven by oncogenic KRAS, which contributes to the production of the reducing agent NADPH [34, 129]. These studies together raise the possibility that non-canonical glutamine metabolism is spatially distributed in tumours depending on rates of lactate production and acidity. A separate study implicates a hypoxia-inducible mitochondria-targeted ASCT2 variant in PDAC metabolism [111], suggesting that mitochondrial glutamine uptake and its canonical metabolism in the tricarboxylic acid cycle is also spatially heterogeneous depending on oxygen availability. Interestingly, hypoxic tumour cells not only produce more lactate but also switch from expressing the epithelial marker E-cadherin to the mesenchymal marker N-cadherin [63]. Hypoxia also leads to upregulation of the transcriptional repressor Blimp1 in a subset of murine KP*C cells, giving rise to a transient, highly metastatic phenotype [52], forging a direct link between transcriptional and metabolic heterogeneity.

## Conclusion

PDAC has proven remarkably recalcitrant to strategies targeting global tumour cell proliferation, making it critical to dissect the drivers of tumour heterogeneity. Despite considerable progress in this area, ITH remains a significant challenge in pancreatic cancer and likely contributes to the high morbidity and mortality of the disease. Unlike many other tumour types, which acquire new subclonal driver mutations during tumour evolution, PDAC frequently has multiple clonal driver mutations at its root, with comparably low genetic heterogeneity within the tumour [49]. Instead, other types of ITH, such as the four frameworks described above, enable tumour progression and resistance to treatment. With PDAC set to become the second-leading cause of cancer death worldwide by 2030 [2], a more complete understanding of the drivers of this cancer is essential moving forward. With the advent of increasingly sophisticated models, old concepts are continually being revised to move the field forward.

The recent explosion of experimentally tractable human primary cell systems, such as patient-derived organoids [130, 131] and PDX models [47], is opening the field to testing key concepts in human-relevant systems. These models have already been employed to study human tumour heterogeneity across the genetic spectrum [132] as well as intratumour metabolic heterogeneity [105], and CSCs versus non-CSCs [28], as discussed earlier. These models will allow the rigorous testing of concepts gleaned from mouse models in human-relevant systems.

Overall, a better understanding of ITH may lead to better treatment options for patients, either through combinatorial approaches targeting multiple aspects of tumour biology, or by reducing the tumour heterogeneity itself in order to increase chemoresponsiveness. As tools become available to rapidly discover, test and translate new therapeutic strategies, the prospect of truly durable clinical responses may become a reality.

## Data Availability

No data are associated with this article. Figures were created with BioRender.com on The Francis Crick Institute’s Premium Plan.
